# Reuma.pt/vasculitis – the Portuguese vasculitis registry

**DOI:** 10.1186/s13023-020-01381-0

**Published:** 2020-05-05

**Authors:** Cristina Ponte, Nikita Khmelinskii, Vítor Teixeira, Karine Luz, Daniela Peixoto, Marília Rodrigues, Mariana Luís, Lídia Teixeira, Sandra Sousa, Nathalie Madeira, Joana A. Aleixo, Teresa Pedrosa, Sofia Serra, Raquel Campanilho-Marques, Walter Castelão, Ana Cordeiro, Inês Cordeiro, Sílvia Fernandes, Carla Macieira, Pedro Madureira, Armando Malcata, Romana Vieira, Fernando Martins, Graça Sequeira, Jaime C. Branco, Lúcia Costa, José Vaz Patto, José Canas da Silva, José A. Pereira da Silva, Carmo Afonso, Helena Canhão, Maria J. Santos, Raashid A. Luqmani, João E. Fonseca

**Affiliations:** 1 Rheumatology Department, Hospital de Santa Maria - Centro Hospitalar Universitário Lisboa Norte, Centro Académico de Medicina de Lisboa, Lisbon, Portugal; 2 Unidade de Investigação em Reumatologia, Instituto de Medicina Molecular, Faculdade de Medicina, Universidade de Lisboa, Lisbon, Portugal; 3 Rheumatology Department, Universidade Federal do Estado de São Paulo, São Paulo, Brazil; 4 Rheumatology Department, Unidade Local de Saúde do Alto Minho, Ponte de Lima, Portugal; 5 Rheumatology Department, Centro Hospitalar e Universitário de Coimbra, Coimbra, Portugal; 6 Rheumatology Department, Hospital de Santo André - Centro Hospitalar de Leiria, Leiria, Portugal; 7 Rheumatology Department, Hospital Garcia de Orta, Almada, Portugal; 8 Rheumatology Department, Hospital Dr. Nélio Mendonça, Funchal, Portugal; 9 Rheumatology Department, Instituto Português de Reumatologia, Lisbon, Portugal; 10 Rheumatology Department, Centro Hospitalar de São João, Porto, Portugal; 11 Rheumatology Department, Centro Hospitalar de Vila Nova de Gaia/Espinho, Gaia, Portugal; 12 Rheumatology Department, Hospital de Egas Moniz, Centro Hospitalar Lisboa Ocidental, Lisbon, Portugal; 13 Multidisciplinary Unit of Chronic Pain, Centro Hospitalar de Setúbal, Setúbal, Portugal; 14 Rheumatology Department, Centro Hospitalar Barreiro Montijo, Barreiro, Portugal; 15 Sociedade Portuguesa de Reumatologia, Lisbon, Portugal; 16 Rheumatology Department, Hospital de Faro, Centro Hospitalar do Algarve, Faro, Portugal; 17 CEDOC, EpiDoC Unit, NOVA Medical School, Universidade Nova de Lisboa, Lisbon, Portugal; 18 Nuffield Department of Orthopaedics, Rheumatology and Musculoskeletal Sciences, University of Oxford, Oxford, UK

**Keywords:** Rare diseases, Vasculitis, Patient registries, Database management systems, Patient reported outcome measures

## Abstract

**Background:**

The vasculitides are a group of rare diseases with different manifestations and outcomes. New therapeutic options have led to the need for long-term registries. The Rheumatic Diseases Portuguese Register, Reuma.pt, is a web-based electronic clinical record, created in 2008, which currently includes specific modules for 12 diseases and > 20,000 patients registered from 79 rheumatology centres. On October 2014, a dedicated module for vasculitis was created as part of the European Vasculitis Society collaborative network, enabling prospective collection and central storage of encrypted data from patients with this condition. All Portuguese rheumatology centres were invited to participate. Data regarding demographics, diagnosis, classification criteria, assessment tools, and treatment were collected. We aim to describe the structure of Reuma.pt/vasculitis and characterize the patients registered since its development.

**Results:**

A total of 687 patients, with 1945 visits, from 13 centres were registered; mean age was 53.4 ± 19.3 years at last visit and 68.7% were females. The most common diagnoses were Behçet’s disease (BD) (42.5%) and giant cell arteritis (GCA) (17.8%). Patients with BD met the International Study Group criteria and the International Criteria for BD in 85.3 and 97.2% of cases, respectively. Within the most common small- and medium-vessel vasculitides registered, median [interquartile range] Birmingham Vasculitis Activity Score (BVAS) at first visit was highest in patients with ANCA-associated vasculitis (AAV) (17.0 [12.0]); there were no differences in the proportion of patients with AAV or polyarteritis nodosa who relapsed (BVAS≥1) or had a major relapse (≥1 major BVAS item) during prospective assessment (*p* = 1.00, *p* = 0.479). Biologic treatment was prescribed in 0.8% of patients with GCA, 26.7% of patients with AAV, and 7.6% of patients with BD. There were 34 (4.9%) deaths reported.

**Conclusions:**

Reuma.pt/vasculitis is a bespoke web-based registry adapted for routine care of patients with this form of rare and complex diseases, allowing an efficient data-repository at a national level with the potential to link with other international databases. It facilitates research, trials recruitment, service planning and benchmarking.

## Introduction

The vasculitides are a group of relatively uncommon and complex diseases. The increased clinical trial activity and new therapeutic options for patients with vasculitis have led to the development of long-term specific registries for this condition [1, 2].

The Rheumatic Diseases Portuguese Register, Reuma.pt, was created in June 2008 with the aim of prospectively record clinical data and treatment adverse effects of patients from all Portuguese rheumatology departments [3–5]. It has been available as a web based online system since 2012 (www.reuma.pt) and currently includes specific modules for twelve different groups of rheumatic diseases (autoinflammatory syndromes, early arthritis, juvenile idiopathic arthritis, myositis, osteoarthritis, psoriatic arthritis, rheumatoid arthritis, scleroderma, Sjögren’s syndrome, spondyloarthritis, systemic lupus erythematosus and vasculitis) in Portuguese and English language. Over 20,000 patients and 170,000 visits have been registered, up to December 2018, by 79 national and international rheumatology centres. On October 2014, Reuma.pt launched a dedicated module to register patients with vasculitis. The variables included were chosen based on other pre-existing European registries, particularly UKIVAS (UK and Ireland Vasculitis Registry) [6], as part of the European Vasculitis Society (EUVAS) collaborative network with the long-term goal of having compatible registries capable of analysing important outcomes in a larger scale [7, 8].

Our aim is to describe the structure of Reuma.pt/vasculitis and give a brief overview on the characteristics of patients registered since its development.

## Methods

### Description and contents

Reuma.pt works as an electronic medical record, which enables prospective collection and central storage of encrypted data. The database information security was approved by the Portuguese National Commission for Data Protection and the ethics committees of the participating institutions. Registered patients are required to sign an informed consent. All identifiable data is encrypted, only accessible through an individual password attributed to clinicians, who can only visualize data related to their centre. Reuma.pt is managed by the Portuguese Society of Rheumatology and has similar structured modules for each disease. Reuma.pt users can work indifferently in Portuguese and English, as the all system is bilingual. A detailed description of its general design and data management has been published elsewhere [3].

**Fig. 1:**
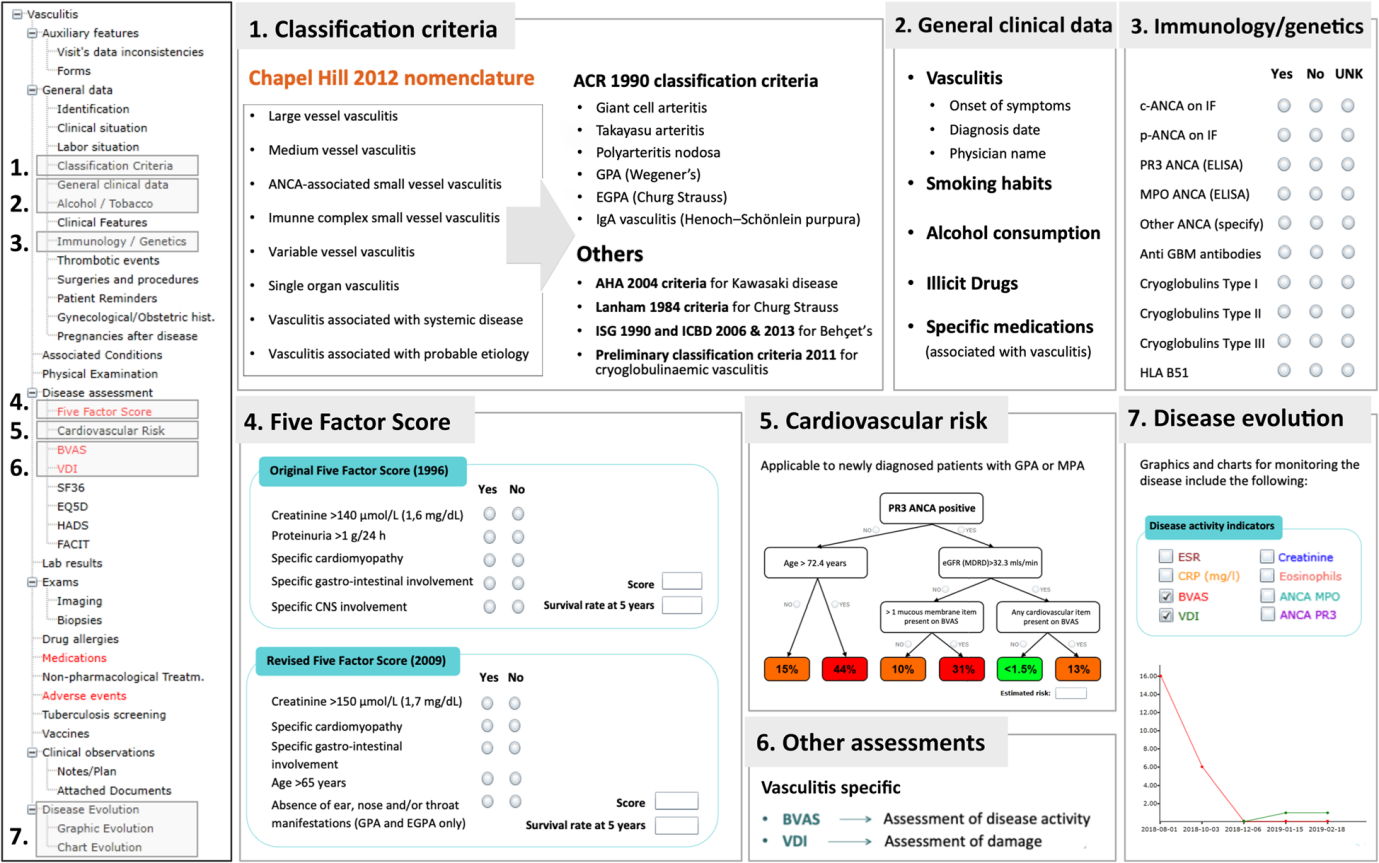
Brief summary of the Reuma.pt/vasculitis contents. *ACR* American College of Rheumatology, *AHA* American Heart Association, *ANCA* anti-neutrophil cytoplasmic antibody, *BVAS* Birmingham Vasculitis Activity Score, *CNS* Central Nervous System, *CRP* C-reactive protein, *eGFR (MDRD)* estimated glomerular filtration rate by the modification of diet in renal disease study equation, *EGPA* eosinophilic granulomatosis with polyangiitis, *ELISA* Enzyme-Linked Immunosorbent Assay, *EQ-5D* EuroQol-5D, *ESR* erythrocyte sedimentation rate, *FACIT* Functional Assessment of Chronic Illness Therapy Fatigue Scale, *FFS* Five Factor Score, *GBM* glomerular basement membrane, *GPA* granulomatosis with polyangiitis, *HADS* Hospital Anxiety and Depression Scale, *HLA* human leukocyte antigen, *ICBD* International Criteria for Behçet’s disease, *IF* immunofluorescence, *ISG* International Study Group, *MPO* myeloperoxidase, *PR3* Proteinase 3, *SF-36* Short form 36, *UNK* unknown, *VDI* Vasculitis Damage Index

The Reuma.pt/vasculitis displays a tree format table of contents on the left hand side (Fig. 1) where standard items, equal to all Reuma.pt modules, and disease specific items are collected. All sections highlighted in red are mandatory for completion at each patient visit. Standard items include patient identification, biobank code for linkage, informed consent status, demographics (date of birth, gender, ethnicity, marital status, education and working status), date of disease onset and diagnosis, past medical history, previous surgeries, smoking and alcohol habits, vaccination, screening for tuberculosis, diagnostic test results, quality of life assessments (Short form 36 [SF-36], EuroQol-5D [EQ-5D], Functional Assessment of Chronic Illness Therapy [FACIT] Fatigue Scale, and Hospital Anxiety and Depression Scale [HADS]), treatment history and report of adverse advents (which is electronically linked to the National Authority of Medicines and Health Products - INFARMED). In addition, patients have their own dedicated area that can also be accessed online to complete the patient reported outcomes before the medical visit.

Within the disease specific items, the 2012 Revised International Chapel Hill Consensus Conference Nomenclature of Vasculitides [9] is used to select the diagnosis subtype (Fig. 1**,** section 1), according to which a possible classification criteria set is available for completion: the 1990 American College of Rheumatology (ACR) classification criteria for giant cell arteritis (GCA, former temporal arteritis) [10], Takayasu’s arteritis (TAK) [11], polyarteritis nodosa (PAN) [12], granulomatosis with polyangiitis (GPA, former Wegener’s granulomatosis) [13], eosinophilic granulomatosis with polyangiitis (EGPA, former Churg-Strauss Syndrome) [14] and IgA vasculitis (IgAV, former Henoch–Schönlein purpura) [15]; the 1984 Lanham criteria also for EGPA [16]; the 2004 American Heart Association Diagnostic Criteria for Kawasaki disease (KD) [17]; the 1990 International Study Group criteria and the 2006 and 2013 International Criteria for Behçet’s disease (BD) [18–20] and the 2011 preliminary classification criteria for cryoglobulinaemic vasculitis (CV) [21]. After the completion of the criteria an automatic sentence appears at the bottom of the screen informing the submitting physician if the patients meets the criteria (example for GCA in Supplementary Fig 1). We expect to update these criteria after the results from the DCVAS study (Diagnostic and Classification Criteria for Vasculitis) [22] are published. Additional information regarding symptoms and signs, which may have not been collected in the classification criteria, are available for completion in a different section - clinical features section - with automatic exportation of data to equivalent items in the first Birmingham Vasculitis Activity Score (BVAS) assessment (Fig. 1, section 6). Moreover, in the general clinical data section (Fig. 1, section 2), specific medications and illicit drugs known to be associated with the development of vasculitis, were extracted from the DCVAS case report form (CRF) and are inquired in this registry. Given the items collected in the DCVAS CRF were revised and agreed upon in a EUVAS meeting in 2010, they work as references for data collection in some European registries (e.g. UKIVAS). Data on specific vasculitis immunology tests (anti-neutrophil cytoplasmic antibodies [ANCA], anti–glomerular basement membrane [anti-GBM] and cryoglobulins), genetics (human leukocyte antigen [HLA]-B51) (Fig. 1**,** section 3) and biopsy features (based on the DCVAS CRF) are also collected. Regarding specific disease assessments: for prognosis the Five Factor score (FFS) - original and revised - is collected for ANCA-associated vasculitides (AAV) and PAN (Fig. 1**,** section 4) [23, 24] and the cardiovascular risk score is available to estimate risk in GPA and microscopic polyangiitis (MPA) [25] (Fig. 1**,** section 5); for disease activity the BVAS (version 3) [26] is used and for damage the Vasculitis Damage Index (VDI) [27] (Fig. 1, section 6). The disease evolution is presented in the form of graphics and charts and includes all measurements available during visits for erythrocyte sedimentation rate (ESR), C-reactive protein (CRP), creatinine, eosinophil count, BVAS, VDI and ANCA titres (Fig. 1**,** section 7).

### Eligibility

Eligible patients include all patients with a diagnosis of vasculitis. Although the structure of Reuma.pt/vasculitis is currently better suited to collect data from adult patients, we are also including patients aged below 18 years. A specific module for Paediatric vasculitis, including the EULAR/PRINTO/PRES criteria [28] and disease specific assessment scores (for e.g. the Paediatric Vasculitis Activity Score [29] and the Paediatric Vasculitis Damage Index [30]), is presently being developed. It will work as a “sub-module” within the general Reuma.pt/vasculitis protocol, into which all paediatric vasculitic patients will “migrate” after being operational. Of interest, Reuma.pt currently has modules for Juvenile Idiopathic Arthritis and other Paediatric rheumatic conditions and so has already extensive functionalities applied to Paediatric rheumatology. In addition, patients with vasculitis secondary to a connective tissue disease are not included in Reuma.pt/vasculitis if their main diagnosis is a disease that already has a Reuma.pt specific module (e.g. systemic lupus erythematosus, rheumatoid arthritis and Sjögren’s syndrome). In those cases, the clinical feature “vasculitis” is available for selection in the different modules, allowing future identification and easy extraction of data if needed. However, only patients registered in Reuma.pt/vasculitis could be evaluated using vasculitis specific assessment tools such as BVAS or VDI. Patients can be registered either at disease presentation or during follow-up, in which case data related to disease onset was registered retrospectively.

### Implementation and recruitment

All centres that work with Reuma.pt are allowed to register patients into Reuma.pt/vasculitis. Before its official launch, all Portuguese Rheumatology centres were invited to participate in the active registry of patients with vasculitis under the care of their department. On the 31st of January 2015, a vasculitis workshop was held in which the first results were presented and strategies to improve the registry of patients were discussed. Training and certification in performing BVAS and VDI was also given at the workshop to ensure correct collection of prospective data.

### Analysis

We analysed all patients registered in Reuma.pt/vasculitis, up to July 2018, with descriptive analysis of the demographics; number of visits per patient; disease duration; diagnosis according to the Chapel Hill nomenclature; classification criteria of the two most common types of vasculitis registered; type of organ involvement; immunology and genetics; imaging and biopsies results (when applicable); immunosuppressive treatment; adverse events; disease specific assessment tools; patient reported outcomes; and follow-up. Regarding the disease specific assessment tools, we assessed the proportion of patients with AAV and PAN who had a FFS of 0, 1 and ≥ 2, and calculated the median and interquartile range (IQR) BVAS at first visit and VDI at last visit for patients with the most common small- and medium-vessel vasculitides recorded. Differences in median BVAS and VDI between vasculitides were compared using Mann Whitney U tests. Patients who had longitudinal data on BVAS recorded, were assessed for the occurrence of any relapses (BVAS ≥1) as well as for major relapses (≥1 major BVAS item) [31]. The number of patients who relapsed within each subtype of vasculitis was compared using chi-square analyses. An adjusted Cox proportional hazard model was used to identify variables affecting survival. Statistical significance was set at *p* < 0.05.

## Results

### General data

A total of 687 patients from 13 different Portuguese centres were registered into Reuma.pt/vasculitis. The number of participating centres increased with time, but around half the patients registered came from an unique centre that has a dedicated vasculitis outpatient clinic [32]. Data was prospectively collected at each visit and in total there were 1945 visits registered (2.8 visits/patient). The mean age was 53.4 ± 19.3 years at last visit; 68.7% were females. The diagnoses registered according to the 2012 Chapel Hill Consensus nomenclature are presented in Table 1. The main features of the most common subtypes of vasculitis registered (n ≥ 20) are presented in Table 2**.** A longer delay in diagnosis was most commonly seen in patients with Behçet’s disease (BD), with an average of 82.5 ± 110.1 (0–672) months from disease onset to diagnosis (disease onset was commonly considered by the physicians as the first disease manifestation attributable to vasculitis); the subtype of vasculitis with less time taken to diagnose since disease onset was GCA with an average of 4 ± 10.9 (0–84) months.

**Table 1:** Diagnosis according the 2012 Revised International Chapel Hill Consensus Conference Nomenclature of Vasculitides

Sub-type of vasculitis	N (%)	
**Large vessel vasculitis**		
Takayasu’s arteritis	28	(4)
Giant cell arteritis	125	(17.8)
Non-classifiable large vessel vasculitis	10	(1.4)
**Medium vessel vasculitis**		
Polyarteritis nodosa^b^	26	(3.7)
Kawasaki disease	1	(0.1)
Non-classifiable medium vessel vasculitis	2	(0.3)
**Small vessel vasculitis**		
**Anti-neutrophil cytoplasmic antibody (ANCA)-associated vasculitis**		
Microscopic polyangiitis	22	(3.1)
Granulomatosis with polyangiitis	41	(5.8)
Eosinophilic granulomatosis with polyangiitis	29	(4.1)
Non-classifiable ANCA-associated vasculitis	11	(1.6)
**Immune complex small vessel vasculitis**		
Cryoglobulinaemic vasculitis	20	(2.9)
IgA vasculitis	16	(2.3)
**Variable vessel vasculitis**		
Behçet’s disease	298	(42.5)
Cogan’s syndrome	12	(1.7)
**Single-organ vasculitis**		
Cutaneous leukocytoclastic angiitis	16	(2.3)
Cutaneous arteritis^b^	4	(0.6)
Primary central nervous system vasculitis	3	(0.4)
Other	10	(1.4)
**Vasculitis associated with systemic disease**		
Rheumatoid vasculitis	2	(0.3)
Other	11	(1.6)
**Vasculitis associated with probable etiology**		
Hepatitis C virus-associated cryoglobulinaemic vasculitis	5	(0.7)
Hepatitis B virus-associated vasculitis	1	(0.1)
Drug-associated immune complex vasculitis	1	(0.1)
Cancer-associated vasculitis	2	(0.3)
Other	5	(0.7)
**TOTAL**^a^	**701**	**(100)**

^a^ The number of diagnoses is higher than the number of patients, given that in 14 cases more than one diagnosis was selected: six patients with cryoglobulinaemic vasculitis and hepatitis C virus-associated cryoglobulinaemic vasculitis (*n* = 5) or other vasculitis associated with systemic disease (*n* = 1); three patients with cutaneous leukocytoclastic angiitis and other vasculitis associated with systemic disease (*n* = 2) or non-classifiable large vessel vasculitis (*n* = 1); two patients with single-organ vasculitis and granulomatosis with polyangiitis (*n* = 1) or other vasculitis associated with systemic disease (*n* = 1); two patients with other vasculitis associated with systemic disease and polyarteritis nodosa (*n* = 1) or non-classifiable ANCA-associated vasculitis (*n* = 1); and one patient with Takayasu‘s arteritis and Hepatitis B virus-associated vasculitis

^b^All patients with polyarteritis nodosa had systemic involvement of the disease; all patients with cutaneous arteritis had limited forms of polyarteritis nodosa

**Table 2:** Demographics, organ involvement, disease assessments and treatment of the most common subtypes of vasculitis registered in Reuma.pt/vasculitis

		TAK	GCA	PAN	MPA	GPA	EGPA	CV	BD
Number of patients (N)		28	125	26	22	41	29	20	298
Demographics									
Female/male ratio		6	1.8	0.9	3.4	1.6	2.2	4	3
Mean age at onset, years (SD)		32.1	74.4	40.4	59.8	47.6	48.1	57.8	27
		(13.8)	(8)	(20.5)	(12.5)	(14.8)	(16.3)	(12.8)	(12.7)
Mean age at diagnosis, years (SD)		34.5	74.7	42.5	60.7	50	51.7	58.7	33.8
		(14.6)	(7.8)	(18.9)	(12.3)	(14.1)	(14.9)	(13.6)	(12.1)
Mean age at last visit, years (SD)		40	77.6	55.8	62.3	56.4	57.6	59.4	43.9
		(15.8)	(7.9)	(18.1)	(15.2)	(12.1)	(15.5)	(15.3)	(14.9)
Organ involvement (%)									
Constitutional symptoms		56.5	59.5	62.5	60.0	69.4	45.8	47.1	26.6
Musculoskeletal		34.8	60.7	79.2	70.0	65.7	54.2	76.5	60.2
Skin		4.5	1.7	75.0	30.0	47.2	60.0	75.0	74.3
Eyes		21.7	47.1	12.5	10.0	31.4	16.7	11.8	36.8
ENT		8.7	50.0	8.7	30.0	82.9	62.5	11.8	4.5
Chest / pulmonary		9.1	1.7	20.8	45.0	58.3	100	–	4.5
Cardiovascular		87.5	69.7	50.0	10.0	8.6	29.2	17.6	17.3
Gastrointestinal		29.2	4.9	20.8	20.0	17.1	16.7	11.8	98.5
Oral ulceration		4.2	–	–	15.0	8.6	8.3	–	98.5
Other symptoms		25.0	4.9	20.8	5.0	8.6	8.3	11.8	16.4
Genitourinary		52.2	3.3	33.3	80.0	58.3	12.5	5.9	83.6
Genital ulceration		4.3	–	–	–	–	–	–	83.6
Other symptoms		47.8	3.3	33.3	80.0	58.3	12.5	5.9	4.0
Neurologic		54.4	88.6	45.8	40.0	42.9	66.7	47.1	19.0
Diagnostic tests									
c-ANCA or ANCA-PR3 positivity N/N of patients (%)		0/14	0/36	0/21	4/21	26/36	3/23	0/15	0/93
		(0.0)	(0.0)	(0.0)	(19.0)	(72.2)	(13.0)	(0.0)	(0.0)
p-ANCA or ANCA-MPO positivity N/N of patients (%)		0/14	0/36	0/21	16/21	6/36	10/23	0/15	1/92
		(0.0)	(0.0)	(0.0)	(76.2)	(16.7)	(43.5)	(0.0)	(1.1)
Vascular imaging with vasculitis ^a^ N/N of patients (%)		22/22	85/95	4/5	−/−	−/−	−/−	−/−	5/8
		(100)	(89.5)	(80.0)					(62.5)
Biopsy compatible with vasculitis ^b^ N/N of patients (%)		2/3	47/72	16/22	6/8	7/21	6/11	1/1	−/−
		(66.6)	(65.3)	(72.3)	(75.0)	(33.3)	(54.5)	(100)	
Disease assessments									
FFS 1996 N/N of patients (%)	FFS = 0	–	–	16/23	7/20	–	17/23	–	–
				(69.6)	(35.0)		(73.9)		
	FFS = 1			5/23	5/20		6/23		
				(21.7)	(25.0)		(26.1)		
	FFS ≥ 2			2/23	8/20		0/23		
				(8.7)	(40.0)		(0.0)		
FFS 2011 N/N of patients (%)	FFS = 0	–	–	18/23	6/20	18/36	10/24	–	–
				(78.3)	(30.0)	(50.0)	(41.7)		
	FFS = 1			4/23	9/20	13/36	13/24		
				(17.4)	(45.0)	(36.1)	(54.2)		
	FFS ≥ 2			1/23	5/20	5/36	1/24		
				(4.3)	(25.0)	(13.9)	(4.1)		
Median BVAS v3 at first visit (IQR)		–	–	12.0	17.5	17.0	15.0	8.0	–
				(6.0)	(9.0)	(15.5)	(12.0)	(10.5)	
Median VDI at first visit (IQR)		–	–	3.0	3.0	1.5	3.0	1.0	–
				(3.0)	(3.0)	(2.8)	(3.0)	(3.0)	
Treatment with DMARDs (%)									
Synthetic DMARDs									
Azathioprine		16.0	0.8	70.8	68.4	41.0	39.3	22.2	30.3
Colchicine		–	–	–	–	.	–	5.6	75.3
Cyclophosphamide oral		–	–	8.3	5.3	23.1	–	5.6	0.8
Cyclophosphamide IV		16.0	0.8	41.7	42.1	41.0	17.9	11.1	5.2
Cyclosporine		–	–	–	5.3	2.6	–	16.7	12.7
Hydroxychloroquine		–	–	4.2	–	5.1	–	16.7	2.0
Leflunomide		–	–	–	–	–	–	5.6	–
Methotrexate		64.0	36.1	33.3	10.5	43.6	17.9	16.7	10.0
Mycophenolate mofetil		–	–	8.3	21.1	5.1	3.6	5.6	0.8
Sulfasalazine		–	–	–	–	–	–	–	4.8
Biological DMARDs									
Adalimumab		–	–	–	–	–	–	–	2.4
Etanercept		–	–	–	–	–	–	–	1.2
Infliximab		4.0	–	8.3	–	–	–	–	5.6
Mepolizumab		–	–	–	–	–	3.6	–	–
Rituximab		–	–	8.3	26.3	41.0	3.6	16.7	–
Tocilizumab		12.0	0.8	–	–	–	–	–	0.4

*ANCA* Anti-neutrophil cytoplasmic antibody, *BD* Behçet’s disease, *BVAS v3* Birmingham Vasculitis Activity Score version 3, *c-ANCA* Cytoplasmic ANCA:*CV* Cryoglobulinaemic vasculitis, *DMARDs* Disease-modifying anti-rheumatic drugs, *EGPA* Eosinophilic granulomatosis with polyangiitis, *ENT* Ear nose and throat, *FFS* Five Factor Score, *GCA* Giant cell arteritis, *GPA* Granulomatosis with polyangiitis, *IQR* Interquartile range, *IV* Intravenous, *LVV* Large vessel vasculitis, *MPA* Microscopic polyangiitis, *MPO* Myeloperoxidase, *PAN* Polyarteritis nodosa, *p-ANCA* Perinuclear ANCA, *PR3* Proteinase 3, *SD* Standard deviation, *TAK* Takayasu’s arteritis, *VDI* Vasculitis Damage Index

^a^In LVV, ultrasound showing halo sign, angiography showing vascular wall thickening, enhancement, stenosis, occlusion, or aneurysms, or PET showing FDG uptake of the aorta or its major branches. In PAN, angiography showing multiple microaneurysms. In BD, angiography showing thrombosis, vascular wall thickening, enhancement, stenosis, occlusion, or aneurysms

^b^In LVV, presence of arteritis, often granulomatous, affecting the aorta or its major branches. In PAN, necrotizing arteritis of medium or small arteries. In AAV, necrotizing vasculitis predominantly affecting small vessels: in EGPA eosinophil-rich and necrotizing granulomatous inflammation, in GPA necrotizing granulomatous inflammation, and in MPA necrotizing inflammation with absence of granulomas. In CV, vasculitis affecting small vessels with cryoglobulin immune deposits

### Classification criteria and diagnostic modalities

The most common diagnoses were Behçet’s disease (BD) (*n* = 298; 42.5%) and giant cell arteritis (GCA) (*n* = 125; 17.8%). All diagnoses recorded were based on physician’s clinical judgement. For BD, 216 patients had enough data to assess the International Study Group (ISG) 1990 criteria and for 251 patients, we could apply the International Criteria for BD (ICBD) (most of the remaining patients were lacking evidence of the results of the pathergy test); 85.3% met the 1990 ISG criteria, 97.2% the 2006 ICBD, and 97.2% the 2013 ICBD. HLA-B51 typing was available in around half the BD cases (*n* = 131), with positive results in 45% (*n* = 59). For GCA, 118 patients had enough data to assess the 1990 American College of Rheumatology classification criteria (most of the remaining patients were lacking biopsy); 97.5% of 118 cases met the criteria. A biopsy of the temporal artery was performed in around half the cases (*n* = 72), compatible with vasculitis in 65% (*n* = 47). Results for ultrasound of the temporal ± axillary arteries were available in 94 cases, 87% showing a non-compressible halo compatible with GCA (*n* = 82). A total of 52 patients with the diagnosis of GCA undertook both exams: 56% (*n* = 29) had positive biopsy and ultrasound; 21% (*n* = 11) had positive ultrasound and negative biopsy; 8% (*n* = 4) had positive biopsy and negative ultrasound; and 15% (*n* = 8) had negative biopsy and ultrasound. Recently, a tendency has been observed for performing ultrasound, rather than biopsy, to diagnose patients with GCA, as well as to complement the ultrasound assessment of temporal arteries with the evaluation of axillary arteries. Detailed description on axillary assessment was available in 36/94 ultrasound reports, with 11/36 (30.5%) cases compatible with large vessel-GCA (LV-GCA). Moreover, a positron emission tomography (PET) was performed to diagnose vasculitis in five patients, revealing LV-GCA in all of them, a computed tomography angiography (CTA) was performed in six patients, compatible with LV-GCA in only two cases, and no patient with GCA was reported to undergo magnetic resonance angiography (MRA). In total, imaging assessment for large vessel involvement of the disease was reported in 41/125 patients with GCA, 16/42 (38.1%) compatible with LV-GCA. In TAK, 36 detailed imaging reports were available for 22/29 patients (79%) with this diagnosis. Results were compatible with vasculitis in 16/16 CTAs, 8/12 PETs, 4/4 catheter-based dye angiograms, 3/3 ultrasounds, and 1/1 MRA. All 22 patients had at least one imaging modality with reported vasculitis.

In other less common subtypes of vasculitis registered, particularly in small vessel vasculitis, the diagnosis was mostly based on immunology and biopsy results. ANCA testing was available in 87.0% of patients with AAV and 80.8% of patients with PAN; Table 2 shows respective ANCA status and subtype. Biopsy results were available in 84.6, 58.5, 37.9 and 36.4% of patients with PAN, GPA, EGPA and MPA, respectively. Table 3 shows the number of biopsies obtained from different sites and percentage of cases compatible with vasculitis. In patients with the diagnosis of cryoglobulinaemic vasculitis, the cryoglobulins subtype was available in 60% of cases (*n* = 12): type II was the most common subtype (58.3%), followed by type I (25.0%) and type III (16.7%). In patients with associated hepatitis C infection, 80% were type II (*n* = 4) and 20% were type I (*n* = 1).

**Table 3:** Biopsies compatible with vasculitis performed in patients with the diagnosis of polyarteritis nodosa or ANCA-associated vasculitides per site of involvement

	PAN	GPA	EGPA	MPA
Patients who underwent biopsy (N)	22	21	11	8
**Positive biopsies per site**^a^**(N/N total performed) (%)**				
• **Ear, nose and throat (ENT)**	–	2/12 (16.7)	1/3 (33.3)	1/3 (33.3)
• **Kidney**	–	3/8 (37.5)	–	6/8 (75.0)
• **Lung**	–	0/6 (0.0)	1/2 (50.0)	–
• **Skin**	13/16 (81.3)	1/2 (50.0)	3/8 (37.5)	–
• **Peripheral nerve**	1/1 (100)	1/3 (33.3)	2/2 (100)	2/2 (100)
• **Intestine**	1/1 (100)	–	–	–
• **Other**	2/7 (28.6)	0/1 (0.0)	0/1 (0.0)	–

^a^ Compatible with vasculitis

*EGPA* Eosinophilic granulomatosis with polyangiitis, *GPA* Granulomatosis with polyangiitis, *MPA* Microscopic polyangiitis, *PAN* Polyarteritis nodosa

### Disease assessment, treatment, and follow-up

Disease assessment in patients with vasculitis targets four main domains - activity, damage, prognosis and quality of life - and it is based on the combination of physical examination, laboratory biomarkers, imaging modalities and assessment tools. In large-vessel vasculitis, given there are no specific monitoring tools, disease assessment is mostly based on clinical symptoms, conventional acute phase markers (erythrocyte sedimentation rate [ESR] and C-reactive protein [CRP]), and imaging. A total of 63 ultrasounds were performed in 35 patients with an established diagnosis of GCA for monitoring purposes.

In small- and medium-vessel vasculitis disease activity is assessed by BVAS and damage by VDI. Although high BVAS and VDI scores have been associated with increased mortality in these patients [33, 34], prognosis is usually assessed using the FFS (FFS 1996 for MPA, EGPA and PAN; and revised FFS 2009 for all AAV and PAN, but not yet validated on non-French patients). Table 2 shows a FFS of ≤1 for most of the AAV and PAN, suggesting that most patients registered are expected to have a good chance of survival at 5 years. Disease activity at first evaluation was higher in patients with AAV, with a median [IQR] BVAS of 17.0 [12.0], in comparison to PAN (BVAS 12.0 [6.0]; *p* = 0.025) and cryoglobulinaemic vasculitis (BVAS 8.0 [10.5]; *p* = 0.002). There were no differences in cumulative damage at last visit between patients with AAV and PAN (median [IQR] VDI 2.0 [3.0] vs. 3.0 [3.0], *p* = 0.847); however, median [IQR] VDI of patients with cryoglobulinaemic vasculitis (1.0 [3.0]) was significantly lower in comparison to PAN (*p* = 0.001) and AAV (*p* < 0.001). In addition, no differences in disease activity at first visit and cumulative damage at last assessment were found amongst each subtype of AAV (*p* > 0.05). Prospective BVAS assessment was available for 12 patients with PAN (collected in 74 visits), 36 patients with AAV (collected in 125 visits) and 2 patients with CV (collected in 5 visits). In PAN, 6/12 (50%) patients had at least one disease relapse recorded (1/6 with a major relapse), in AAV, 18/36 (50%) patients had at least one relapse recorded (7/18 with a major relapse); and in CV, 1/2 (50%) patients had at least one relapse recorded (corresponding to a major relapse). No differences were found in the proportion of patients with a diagnosis of PAN or AAV who relapsed (*p* = 1.00) or had a major relapse (*p* = 0.479). Moreover, amongst each subtype of AAV, there were no differences found in the number of patients who relapsed (MPA 4/10, GPA 8/17, and EGPA 6/9; *p* > 0.05) or who had a major disease relapse (MPA 2/4, GPA 2/8, and EGPA 2/6; *p* > 0.05). In all vasculitides quality of life has been assessed by generic measures such as the SF-36, currently included in the AAV core set measures of assessment endorsed by the Outcome Measures in Rheumatology (OMERACT) Vasculitis Working Group [35] and used in trials of large-vessel vasculitis (LVV) [36]. However, the rate of its completion in Reuma.pt/vasculitis was very low; only 9.6% (*n* = 66) of patients had at least one SF-36 completed in the evaluation of their disease (total of 97 visits), most commonly assessed in BD (*n* = 55), followed by AAV (*n* = 4) and LVV (*n* = 4). Other patient reported outcomes were also evaluated in less then 10% of patients: 67 patients (9.8%) completed FACIT in 89 visits; 64 patients (9.3%) completed HADS in 87 visits; and 43 patients (6.3%) completed EQ-5D in 69 visits.

Data on treatment with disease-modifying antirheumatic drugs (DMARDs) for the most common vasculitides registered is detailed in Table 2**.** A total of 59 biologic DMARDs, most frequently rituximab (*n* = 27; 45.7%), were prescribed for 53 patients. The following proportion of patients within each subtype of vasculitis, in whom detailed therapy was registered, were treated with biologic DMARDs: 4/25 (16.0%) patients with TAK; 1/122 (0.8%) patients with GCA; 3/24 (12.5%) patients with PAN; 5/19 (26.3%) patients with MPA; 16/39 (41.0%) patients with GPA; 2/28 (7.1%) patients with EGPA; 3/18 (16.7%) patients with CV; and 19/251 (7.6%) with BD. Moreover, 77 adverse advents related to treatment (in around ¼ of cases considered severe) were reported in 53 patients, most commonly due to prednisolone (*n* = 18; 23%), followed by methotrexate (*n* = 15; 19%) and azathioprine (*n* = 10; 13%).

Deaths were reported in 34 (4.9%) patients (20 females and 14 males), at a mean age of 72.6 ± 15.5 [30–98] years and mean disease duration of 4.9 ± 5.7 [0–22] years; however, specific cause of death was available in only 10 cases, none of which directly related to treatment. Infection was the most common cause of death (*n* = 4; respiratory infection in three cases and urosepsis in one case), followed by cardiovascular events (*n* = 3; stroke, heart attack, and pulmonary embolism), complications of the disease (*n* = 2; ischaemic colitis in a patient with PAN and complication of an aortic aneurysm surgery in a patient with TAK) and acute kidney injury (*n* = 1). Deaths were most commonly reported in patients with the diagnosis of GCA (*n* = 11), followed by AAV (*n* = 6), CV (*n* = 5), PAN (*n* = 2), TAK (*n* = 2), BD (*n* = 2), and other subtypes of vasculitis (*n* = 6). However, an adjusted Cox proportional hazard model for age and sex, demonstrated that there was no statistically significant difference in terms of survival between patients with the diagnosis of GCA and other vasculitides (HR 0.53, 95% CI: 0.21–1.36), and AAV and other vasculitides (HR 1.08, 95% CI: 0.42–2.79). Patients with AAV who underwent biologic DMARDs presented an increased rate of 5-year mortality (HR 16.44, 95% CI 1.71–157.65), but no differences in survival were found between AAV patients with a FFS of 0 and a FFS of ≥1 (1996 FFS: HR 5.51, 95% CI 0.22–140.17; 2009 FFS: HR 0.69, 95% CI 0.42–11.29). There were three patients discharged from hospital care into general practice due to disease remission (GCA [*n* = 1], BD [*n* = 1], and cutaneous leukocytoclastic angiitis [*n* = 1]) and 43 cases of lost to follow-up (most frequently in patients with BD [*n* = 17] and GCA [*n* = 8]).

## Discussion

Reuma.pt/vasculitis is an efficient electronic clinical record and online registry for patients with the diagnosis of vasculitis, designed to record all relevant data in clinical practice following a standardised approach. It provides an opportunity to improve the quality of clinical care in this group of rare diseases and characterize the natural history of vasculitis captured by different Portuguese Rheumatology centres.

Reuma.pt/vasculitis works as a data repository at a national level with the potential to link with other international databases. To our knowledge, there are eight other European countries with an established vasculitis registry: United Kingdom and Ireland (UKIVAS) [6, 37]; France (FVSG registry) [38]; Spain (REVAS) [39]; Poland (POLVAS registry) [7, 40]; Norway (NorVas) [41]; Czech Republic (Czech Registry of AAV) [42]; and Greece (Greek Registry of AAV) [43]. In addition, in many other European countries the development of a vasculitis registry is on their research agendas (e.g. Germany, Switzerland, the Netherlands and Italy) [8]. Reuma.pt/vasculitis was developed based on local expertise and adaptions from other pre-existing European registries (mainly UKIVAS) and the DCVAS CRF (the largest international study in vasculitis) [22]. Therefore, it allows for compatible future joint exports as part of the EUVAS long-term strategy of aligning the European vasculitis registries and establish agreed core-set items present in all registries [8]. This capability is also an essential requirement of the current European Reference Network initiative to improve care for patients with rare diseases, including vasculitis [44].

Reuma.pt/vasculitis comprises a group of various subtypes of vasculitis, with different clinical manifestations, classification criteria and disease specific assessments and outcomes of interest. In addition, the registry of data by the physicians is done on an entirely voluntary basis. Therefore, balancing granularity and feasibility of data collection, when constructing the database, posed a challenge. Although we encountered missing data in our analysis, mainly on the prospective disease assessments and cause of death, overall our data was robust and allowed us to obtain reliable information on many of our patients’ characteristics. More than 1/3 of patients had the diagnosis of BD (*n* = 298), largely followed by GCA (*n* = 125). Although Reuma.pt/vasculitis is a registry reflecting the reality of the Portuguese Rheumatology centres and is generally not intended for epidemiologic studies, the number of patients with BD registered (97.2% and 85.3% fulfilling the ICBD and the ISG criteria, respectively) exceeded the previously reported prevalence of 1.5 per 100,000 in 1991 [45] and of 2.4 per 100,000 in 1997 [46], highlighting the need for new epidemiologic studies on this disease in Portugal. Another interesting fact is that there were more patients diagnosed with GCA that undertook ultrasound in comparison to TAB (*n* = 94 vs. *n* = 72), reflecting modern practice and the incorporation of ultrasound as an essential diagnostic tool in the Rheumatology setting [47]. The immunosuppressive treatment used in these patients is in line with current European League Against Rheumatism (EULAR) recommendations for the management of AAV, LVV and BD [48–50]; the fact that only one patient with GCA (0.8%) was treated with tocilizumab reflects the low national accessibility to this DMARD in the context of this indication, which is expected to change in the near future.

There were some limitations found in Reuma.pt/vasculitis. Besides the expected missing data and underreporting (e.g. imaging results in LVV and PAN, biopsy results and ANCA status in AAV, detailed treatment, etc.), very frequently encountered in this sort of registry, the diagnosis of each subtype of vasculitis was purely based on the submitting physician’s clinical knowledge and experience, therefore lacking external validation. In addition, most of the classification criteria used are currently outdated and waiting for the DCVAS results to ensure proper update [22]. Moreover, not all physicians who registered patients are either certified on the BVAS and VDI assessments, or have proper practice in evaluating patients with vasculitis using these assessment tools. Although we provided initial training we recommend more practice and certification sessions in the future to guarantee data quality and correct assessment of disease activity and damage, particularly in patients under biologic DMARDs.

We expect this registry to continue expanding, including more centres, different specialties (e.g. Nephrology) and other data items. We aim to include the new AAV patient-reported outcomes (AAV-PRO) [51]; a specific module for Paediatric patients; and potentially divide the registry into a basic dataset (with mandatory collection of data), intermediate dataset (to include more advanced fields on the dataset, such as patient reported outcomes) and advanced dataset (used for specific trials and clinical studies), therefore increasing the feasibility of data collection and reducing the inherit missing data.

## Conclusion

We anticipate that Reuma.pt/vasculitis will form the basis of many future national and international studies of vasculitis by allowing recruitment into trials, benchmarking and service planning. Its link to the biobank will facilitate translational research, and its connection to INFARMED will ensure proper report of adverse effects related to treatment. In addition, Reuma.pt/vasculitis will be an important source for longitudinal observational studies on the most relevant outcome measures of interest in vasculitis (e.g. treatment efficacy).

## Supplementary information

**Additional file 1: Figure S1.** How to register the classification criteria of a patient with giant cell arteritis.

## Data Availability

The datasets used and/or analysed during the current study are available from the corresponding author on reasonable request.

## Abbreviations

AAV: ANCA-associated vasculitis
AAV-PRO: AAV patient-reported outcomes
ACR: American College of Rheumatology
AHA: American Heart Association
ANCA: Anti-neutrophil cytoplasmic antibodies
Anti-GBM: Anti–glomerular basement membrane
BD: Behçet’s disease
BVAS: Birmingham Vasculitis Activity Score
c-ANCA: Cytoplasmic ANCA
CNS: Central Nervous System
CRF: Case report form
CRP: C-reactive protein
CTA: Computed tomography angiography
CV: Cryoglobulinaemic vasculitis
DCVAS: Diagnostic and Classification Criteria for Vasculitis
DMARDs: Disease-modifying antirheumatic drugs
eGFR (MDRD): Estimated glomerular filtration rate by the modification of diet in renal disease study equation
EGPA: Eosinophilic granulomatosis with polyangiitis
ELISA: Enzyme-Linked Immunosorbent Assay
EQ-5D: EuroQol-5D
ENT: Ear nose and throat
EULAR: European League Against Rheumatism
EUVAS: European Vasculitis Society
ESR: Erythrocyte sedimentation rate
FACIT: Functional Assessment of Chronic Illness Therapy Fatigue Scale
FFS: Five factor score
FVSG: French Vasculitis Study Group
GBM: Glomerular basement membrane
GCA: Giant cell arteritis
GPA: Granulomatosis with polyangiitis
HADS: Hospital Anxiety and Depression Scale
HLA: Human leukocyte antigen
HR: Hazard-ratio
ICBD: International Criteria for Behçet’s disease
IF: Immunofluorescence
IgAV: IgA vasculitis
IQR: Interquartile range
INFARMED: National Authority of Medicines and Health Products
ISG: International Study Group
IV: Intravenous
LV-GCA: Large vessel-GCA
LVV: Large vessel vasculitis
MPA: Microscopic polyangiitis
MPO: Myeloperoxidase
MRA: Magnetic resonance angiography
NorVas: Norwegian Vasculitis Registry
OMERACT: Outcome Measures in Rheumatology
p-ANCA: Perinuclear ANCA
PAN: Polyarteritis nodosa
PET: Positron emission tomography
POLVAS: Polish Vasculitis Registry registry
PRES: Paediatric Rheumatology European Society
PR3: Proteinase 3
PRINTO: Paediatric Rheumatology International Trials Organization
REVAS: Spanish Registry of systemic vasculitis
SD: Standard deviation
SF-36: Short form 36
TAK: Takayasu’s arteritis
UKIVAS: UK and Ireland Vasculitis Registry
UNK: Unknown
VDI: Vasculitis Damage Index
KD: Kawasaki disease
