# Supplementary figures and images for: Reuma.pt/vasculitis – the Portuguese vasculitis registry
Source: Orphanet J Rare Dis. 2020 May 5;15:110. doi: 10.1186/s13023-020-01381-0 (PMC7201571; doi:10.1186/s13023-020-01381-0)

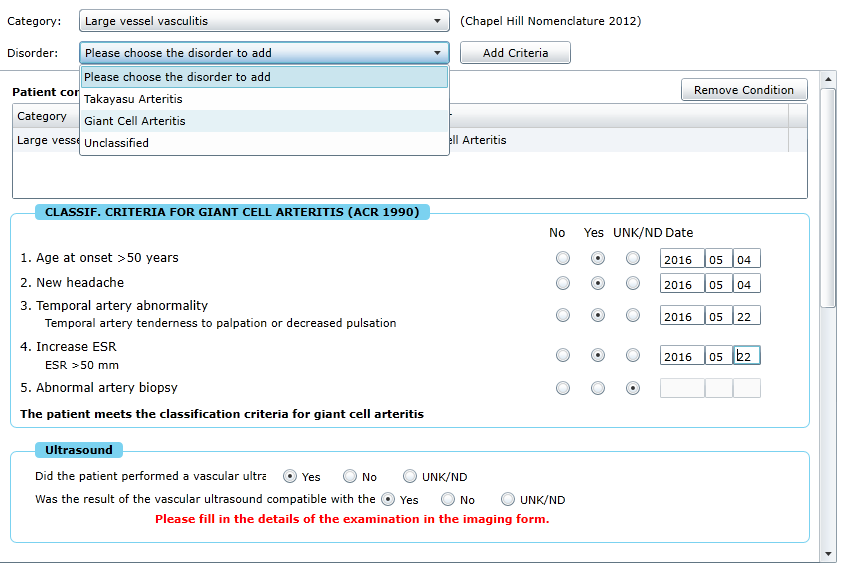

Supplement: Supplementary file 1 — Additional file 1: Figure S1. How to register the classification criteria of a patient with giant cell arteritis. [file 13023_2020_1381_MOESM1_ESM.png]
